# Preferred Provider Relationships Between Medicare Advantage Plans and Skilled Nursing Facilities Reduce Switching Out of Plans: An Observational Analysis

**DOI:** 10.1177/0046958018797412

**Published:** 2018-09-03

**Authors:** Elizabeth M. Goldberg, Laura M. Keohane, Vincent Mor, Amal N. Trivedi, Hye-Young Jung, Momotazur Rahman

**Affiliations:** 1 Brown University, Providence, RI, USA; 2 Vanderbilt University School of Medicine, Nashville, TN, USA; 3 Providence VA Medical Center, RI, USA; 4 Weill Cornell Medical College, New York, NY, USA

**Keywords:** Medicare, Medicare Advantage, skilled nursing facilities, logistic models, federal health insurance plans

## Abstract

Unlike traditional Medicare, Medicare Advantage (MA) plans contract with specific skilled nursing facilities (SNFs). Patients treated in an MA plan’s preferred SNF may benefit from enhanced coordination and have a lower likelihood of switching out of their plan. Using 2011-2014 Medicare enrollment data, the Medicare Healthcare Effectiveness Data and Information Set, and the Minimum Data Set, we examined Medicare enrollees who were newly admitted to SNFs in 2012-2013. We used the Centers for Medicare & Medicaid Services star rating to distinguish between MA plans and show how SNF concentration experienced by patients varies between patients in plans with different star ratings. We found that highly rated MA plans steer their patients to a smaller number of SNFs, and these patients are less likely to switch out of their plans. Strengthening the MA plan–SNF relationship may lower disenrollment rates for SNF beneficiaries, imparting benefits to both patients and payers.

**What do we already know about this topic?**
Seniors who utilize high-cost services, particularly skilled nursing facility (SNF) care, are more likely to leave the Medicare Advantage (MA) program in favor of traditional Medicare.
**How does your research contribute to the field?**
We found that highly rated MA plans steer their patients to a smaller number of SNFs and these patients are less likely to switch out of their plans.
**What are your research’s implications toward theory, practice, or policy?**
Embracing selected SNFs as part of the network of preferred providers may decrease disenrollment rates for SNF beneficiaries and facilitate MA plans’ ability to continue to manage care for high-cost beneficiaries.

## Introduction

For more than 25 years, Medicare beneficiaries have been able to opt for private health insurance plans, currently known as Medicare Advantage (MA) plans. The original rationale for MA plans was that capitated payments to risk-bearing plans would incentivize innovative approaches to improving care and reduce unnecessary health care use. Despite this potential, capitated payments also incentivize enrollment of healthy patients and the avoidance of complex patients, a phenomenon known as “favorable risk selection.” Over the last decade, the MA program grew rapidly. Currently, 33% of all Medicare beneficiaries are enrolled in MA plans and enrollment is projected to increase from 17.6 Million in 2016 to 22 Million by 2020.^1,2^ This rapid expansion of MA was triggered by the Medicare Modernization Act (MMA) of 2003. The main changes under the MMA included the adoption of a new hierarchical chronic condition (HCC)–based payment formula, an annual lock-in provision, and provisions allowing MA plans to steer patients to their preferred set of care providers. Several studies documented that these changes were fairly successful in terms of improving care and reducing favorable risk selection.^3-5^

While MA has been successful in making improvement for the overall population, the performance of MA in delivering care for high-cost Medicare beneficiaries remains questionable. Brown et al^6^ showed that favorable risk selection is higher among beneficiaries with high HCC risk scores because the variation in health care spending is higher among this group. Recent research also showed that beneficiaries who utilize high-cost services, particularly skilled nursing facility (SNF) care, are more likely to leave the MA program in favor of traditional Medicare (TM).^7,8^ This may be in part due to cost-sharing differences across MA plans. Patients with greater health care utilization may be unable to afford to remain in their MA plan due to high out-of-pocket expenses.^9,10^ In 2012, the switching rate to TM was about 3% among all MA enrollees, but was 8% among MA enrollees with a short SNF stay and 15% among MA enrollees with a long SNF stay.^8^ Individuals were considered short-stay SNF users if they had at least one Minimum Data Set (MDS) assessment, but not a quarterly or annual one. Any individual who had a separate short- and long-stay SNF episode in a given year was considered a long-stay SNF user.

While switching may be beneficial to some enrollees—by allowing them to enroll in a plan that better meets their health care needs—switching can also be undesirable. First, patients may switch due to being unsatisfied with the SNF care they recently received. Second, the disproportionate outflow of high-cost beneficiaries from MA to TM presents a net transfer of liabilities from private to public insurance. Finally, conceptually, the benefit of carefully managed care should be higher for high-need, high-cost members than it is for healthier beneficiaries and the gains from managed care could be higher if MA plans were more successful in retaining these beneficiaries. Despite these important implications, we have very little understanding of the factors that drive the exodus of high-cost MA patients to TM.

The goal of this article is to examine whether preferred provider relationships between MA plans and SNFs affect a patient’s likelihood of switching following SNF use. For the purposes of this analysis, switching is defined as disenrolling from an MA contract and subsequently enrolling in a different MA contract or TM. Star ratings and payment adjustments vary by MA contract. An MA contract can offer several different plans with different benefits, but all plans offered by an MA contract have the same star rating. For instance, Blue Cross & Blue Shield of Rhode Island has a contract with Medicare to offer MA plans. These plans may include health maintenance organization (HMO) plans, preferred provider organization (PPO) plans, special needs plans (SNPs), and others. All Blue Cross & Blue Shield of Rhode Island HMO plans have the same star rating, although they differ in premiums, out-of-pocket costs, and whether they cover dental services and other benefits. Unlike TM, many MA plans have a preferred network of care providers.^11^ The MMA permitted this practice because increasing the share of patients from a particular MA plan in an SNF (“high concentration”) may enhance coordination and integration of care through economies of scale. We hypothesize that patients treated in an MA plan’s preferred SNFs will have a lower likelihood of switching. We also examined how MA contracts with different star ratings vary in terms of steering their patients to selected SNFs and switching rates. Medicare assigns star ratings at the contract rather than the individual plan level and high switching rates decrease a contract’s star rating. Therefore, we hypothesize that MA contracts with higher star ratings have higher levels of concentration and lower switching rates.

## Conceptual Framework

Our conceptual framework has two components. First, we describe the incentives of the MA plan and SNF that may influence a patient’s decision to switch plans. Second, we argue that the concentration of patients from a particular MA contract in a particular SNF may align the incentives of both the MA plan and the SNF. Although we define concentration at the MA contract level, we will use the more widely used term *plan* for contract in the subsequent text.

MA plans have both positive and negative incentives to encourage selective disenrollment of SNF patients. On one hand, because MA plans receive capitated payments based on a patient’s HCC score, they have incentives to attract the healthier Medicare beneficiaries within an HCC score group. Brown et al^6^ showed that the variation in health care spending among high HCC beneficiaries is much higher than the variation in care spending among the low HCC score group. Because SNF users are likely to have high HCC scores, the degree of adverse selection is likely to be higher in this population. A SNF stay may increase the expected future costs after adjusting for HCC score and this may encourage MA plans to avoid SNF users. MA plans may steer high-cost patients to lower quality SNFs in an effort to encourage disenrollment and reduce the plan’s future care spending. On the other hand, high switching rates negatively affect MA plan star ratings, which affect payment rates from Medicare and attractiveness to potential enrollees.^12-14^ In addition, the MMA also incorporated a separate payment formula for institutionalized beneficiaries that could encourage MA plans to enroll long-stay SNF residents.

SNFs may be incentivized to encourage their patients to switch to TM because payment rates from TM tend to be higher than payment rates from MA plans. Based on a recent MedPAC report, TM payment rates were about 23% higher than what MA plans reimburse SNFs for similar services.^15^ Another consideration is that SNFs historically only served TM patients and they may be reluctant to adapt to managed care practices, including prior authorization requirements for SNF services.

Given these incentives, we argue that the concentration of patients from a particular MA plan in a particular SNF may align these incentives through economies of scale. The MMA allowed MA plans to steer patients to their preferred set of providers because such concentration theoretically reduces the cost of delivering care through economies of scale. Several studies also argued that economies of scale incentivize MA plans and SNFs to cooperate.^16-19^

Increasing the concentration of residents from a single MA plan in a given SNF theoretically increases the ability of MA clinicians to monitor residents’ conditions efficiently. Because MA plans are liable for their members’ hospitalizations, they are incentivized to closely manage care. They may strive to reduce the need for hospitalization via regular physician or nurse practitioner visits. If a plan has only one member residing in an SNF, devoting those resources to the members’ medical care will be operationally difficult and costly and both the patient and the plan may be happier with disenrollment. Similarly, from the SNFs perspective, because SNFs have historically had difficulties securing committed physicians to manage their TM patients and avoidable hospitalizations are frequent,^20-22^ their interests are aligned with MA plans committed to providing structured medical management. In addition, such arrangements can also ensure a constant flow of new patients to the SNF. Thus, we hypothesize that coordination between the MA plan and SNF reduces the disenrollment of these patients.

A key aspect in a patient’s disenrollment decision is the SNF length of stay. For instance, patients who become long-stay residents as opposed to residents with a short SNF stay benefit more from such coordination (because plan physicians and nurse practitioners are in the building on a regular basis) and are also more likely to comply with the SNF’s preferred insurance. Improved coordination between the patient’s MA plan and SNF is likely to reduce SNF length of stay by increasing efficiency of SNF care. Thus, we also hypothesize that coordination between the MA plan and SNF reduces the likelihood of becoming a long-stay resident among newly admitted SNF patients.

Another important issue is the heterogeneity of MA plans because they vary widely in quality^23^; MA plans may have varying approaches to how they manage and coordinate care with providers. Given the possible benefits of coordination, high-quality MA plans may devote more effort to coordination. On the contrary, given some incentives under which MA plans operate described above, some may choose not to engage in coordination to encourage these high-cost patients to switch plans or to switch to TM. Thus, we hypothesize that MA plans with higher star ratings will have higher levels of concentration and lower switching rates.

## New Contribution

In order to reduce the cost of care delivery health insurance companies frequently steer patients to a preferred set of providers, but little is known about the welfare implications of a preferred provider network on patients. This is the first article, to the best of our knowledge, that examines the effect of such steering on patients’ likelihood of switching, which is a possible indicator of patient satisfaction and quality of care received.

We employ novel strategies to better understand preferred provider networks in MA. There is no source of publicly available information on provider networks in MA, so we use the distribution of admissions to all SNFs in the United States from all MA plans to identify concentration of patients from a particular MA plan in a particular SNF. We document how the practice of steering patients varies between MA plans with different Centers for Medicare & Medicaid Services (CMS) ratings. In addition, by incorporating SNF and MA plan fixed effects, we are able to isolate the effect of concentration while controlling for the quality of the MA plan and the SNF.

## Methods

### Data Sources

This study relies upon three sources of individual-level data. These include the Medicare Enrollment file, Medicare Healthcare Effectiveness Data and Information Set (HEDIS) data and the MDS for SNF resident assessment. These data are linked via an encrypted beneficiary ID and then to a provider ID with match rates exceeding 98%.^24^ We used data for three years: 2012-2014. These data sets are described in the section below.

The Medicare enrollment file contains demographics, date of death, managed care participation (identified monthly), Part D coverage, dual eligibility, and ZIP code of residence. Among Medicare managed care enrollees, information on enrollment in the specific MA plan is available from HEDIS data. It also contains individual-level utilization data for all enrollees in MA plans. CMS requires most MA plans to report these data, including number of hospital admissions, emergency department admissions, and nonacute stays.

The use of SNFs and SNF resident characteristics are available from the national repository of the MDS. The assessments are reported for all patients admitted to Medicare-certified SNFs, including enrollees in both TM and MA. Assessments are done upon admission and quarterly thereafter. Starting in FY2011, CMS mandated the MDS 3.0, which improved the completeness of these data in two ways: fewer missing assessments and fewer missing values in variables. For example, comparing the findings from Mor et al^24^ and Rahman et al,^25^ discharge assessments are more completely recorded under the new version. Similarly, comparing findings of Mor et al^26^ and Wysocki et al,^27^ there is less missing data in the new version.

Besides individual patient-level data, we used two sources of provider-level data. We used MA plan star rating data and plan type data downloaded from the CMS Web site.^28^ Organizational-level data on SNFs were derived from the Online Survey Certification & Reporting System (OSCAR) dataset, which is maintained by CMS to track SNF performance.

### Study Cohort

Our study cohort included Medicare beneficiaries who entered SNFs in 2012 and 2013, who were enrolled in MA on the month of SNF admission and did not have any SNF stays in the one year preceding their date of admission. Only the enrollment status for the index SNF stay was examined in this analysis. The enrollee may have had subsequent SNF stays in the same year. We applied four exclusion criteria. First, we excluded individuals who died in the following twelve months because our objective was to assess plan choice one year after SNF admission. Second, we excluded MA enrollees in plans that did not report HEDIS data (about six percent). Third, we also excluded about 50 000 individuals who were enrolled in SNPs during the month of index SNF admission. Patients who switched into a SNP plan after the index SNF admission month were not excluded. SNPs are a type of MA plan that are available only to people with specific diseases or characteristics, such as patients who are already institutionalized, are dual-eligible, or have a severe chronic condition. For instance, patients who become Medicaid eligible after their SNF stay can switch into a dual-eligible special needs plan (D-SNP). These patients were included in the analysis because they were not enrolled in a SNP plan during the month of index SNF admission. Finally, we excluded enrollees in 2 or 2.5 star rating plans because CMS started urging enrollees in 2012 in these plans to switch.^12,29-31^ Our final sample included 529 962 Medicare beneficiaries enrolled in 390 MA contracts and treated in 13 611 SNFs.

### Variables

We identified baseline enrollment in MA on the month of SNF admission using the Medicare enrollment file. We used the HEDIS file to determine the contract in which MA members were enrolled. We then identified the star rating of each MA contract from CMS data.

To measure our outcome variable—switching—we identified beneficiaries’ enrolled plan twelve months after SNF admission. We used the one-year follow-up period methodology following previous studies on switching.^7,8^ Prior to 2006 MA plan members could switch anytime of the year, but since then the Medicare program instituted an annual lock-in provision. This lock-in provision commits beneficiaries to their plan choice until an annual open enrollment period. Exceptions are made for dual-eligible beneficiaries and nursing home residents. In addition, enrollees can switch to a 5-star plan anytime of the year. Similar to baseline insurance enrollment, we first checked beneficiaries’ MA enrollment in the month after admission from the enrollment file and then identified the MA contract using HEDIS. If a beneficiary switched plans, we distinguished between switching to TM or to another MA contract. Of note, because plan identification within an MA contract may change over time, we did not code within contract switching between MA plans as switching.

We included the following patient characteristics: age, sex, race, and dual eligibility status on the month of SNF admission. These were obtained from the Medicare enrollment file. We also included the activities of daily living (ADL) score, cognitive performance scale (CPS), and several key diagnosis indicators from the MDS data.

Our main explanatory variable is concentration. This is an SNF-MA contract-level variable measured as the share of a SNF’s admissions between 2012 and 2013 (both MA and TM) coming from the patient’s enrolled MA contract. The denominator is the number of all admissions to a SNF from 2012-2013. The numerator is the number of admissions from the MA contract of the patient during 2012-2013. Of note, this variable varies across patients within a SNF depending on the enrolled MA contract and across patients within an MA contract depending on the admitting SNF.

Besides the relationship with a particular SNF, a MA plan might be particularly invested in a county due to economies of scale. SNFs in that county will experience a higher share of patients from this plan. As a result, for a given patient, the concentration in a particular SNF and concentration in a particular county are likely to be correlated and both variables can be associated with switching. So, we calculated an MA plan–county-level control variable, which is the share of a SNF county’s Medicare beneficiaries enrolled in a patient’s MA plan.

### Analysis

The first step of our analyses is to examine variation of the explanatory variable, ie, the share of a SNF’s patients coming from the patient’s enrolled MA plan. If concentration of patients in the SNF from a specific plan is beneficial to patients, high-performing plans may have a higher concentration. Therefore, we used CMS’s star rating to distinguish between MA plans and to test whether SNF concentration experienced by patients varies between patients in plans with different star ratings.

We compared switching rates of patients experiencing different (high vs low) concentration enrolled in MA plans with different star ratings. Though concentration is a continuous variable, we used a cutoff of ten percent (approximately 50th percentile) or greater to define high concentration. Thus, a high-concentration SNF implies that the share of a SNF’s patients enrolled in a patient’s MA plan is greater than or equal to ten percent. These comparisons were adjusted for patient characteristics. To perform the adjustment, we first estimated a logit model of an outcome onto patient characteristics and interactions of a patient’s plan star rating and an indicator of high concentration (ie, whether a patient’s SNF has at least ten percent of its patients enrolled in the patient’s plan). We then calculated the adjusted predicted probabilities of an outcome for the interaction terms using the “margins” command in Stata.^32^ We then plotted the probabilities.

To formally test the relationship between MA concentration within an SNF and an outcome, we estimated the following regression model:

$$\begin{array}{l} Outcome_{inc}=\alpha NH\_concen_{nc}+\beta market\_concen_{nc}\\ \;\;\;\;\;\;\;\;\;\;\;\;\;\;\;\;\;\;\;+X_{i}\gamma +\delta_{n}+\theta_{c}+u_{inc}. \end{array}$$

$Outcome_{inc}$ is a binary outcome variable (switching) experienced by an MA beneficiary enrolled in contract *c* and admitted to SNF *n*. We used three types of switching outcomes: switching to another MA contract, switching to TM, and any switching (either to another MA or to TM). $NH\_concen_{nc}$ is our main explanatory variable: the share of SNF *n*’s patients coming from the patient’s MA contract *c*. $market\_concen_{nc}$ is the share of Medicare beneficiaries in SNF *n*’s county enrolled in MA contract *c*. $X_{i}$ is a vector of patient characteristics obtained from the enrollment file and SNF admission assessments. $\theta_{c}$ reflects MA contract fixed effects and $\delta_{n}$ reflects SNF fixed effects. We used a linear probability model to estimate this regression.

α is our parameter of interest that measures change in the outcome associated with a one percentage point change in the share of an SNF’s patients enrolled in a patient’s MA plan. There is tremendous variation in quality, payer mix, and cost factors across SNFs, and MA plans can negotiate prices with SNFs. $\delta_{n}$, SNF fixed effects, capture all these observed and unobserved SNF characteristics. On the contrary, MA plans vary widely in terms of size and quality. $\theta_{c}$ captures the observed and unobserved MA plan characteristics. Thus, this specification assumes that MA plan concentration in a particular SNF has an effect on the outcome in addition to the MA plan’s and SNF’s own effect.

The effect of concentration can be nonlinear. To test the nonlinearity of the effect, we categorized $NH\_concen_{nc}$ into three categories: $NH\_concen_{nc}$<10%, 10% ⩽ $NH\_concen_{nc}$<20%, $NH\_concen_{nc}$ ⩾ 20%. We used ten percent and twenty percent as thresholds because they were approximately the 50th percentile and 75th percentile of concentration experienced by patients in our sample. We used the first category as the reference group and used two binary indicators for the two remaining categories. We estimated the above specified model replacing the continuous version of $NH\_concen_{nc}$ with its categorical version. We also estimated this regression model for several subsamples. First, we estimated our model separately for enrollees in low (3, 3.5, and 4) star and high (4.5 and 5) star MA plans (the lowest quality plans were not included in this analysis). Second, we ran separate analyses for short-stay and long-stay SNF patients. If a patient stays in an SNF for a very short period of time, he or she may not benefit from concentration. Thus, the influence of concentration is expected to be greater on long-stay residents than those who stayed in the SNF for a short period of time. We categorized beneficiaries as long-stay if the individual stayed in an SNF for more than 100 days in the six months following SNF admission.^33-35^ Finally, we performed separate regression analyses for dual- and non–dual-eligible patients because dual-eligible patients are allowed to switch any time of the year and have higher switching rates.

## Results

We included 529 962 MA beneficiaries who were newly admitted to SNFs in 2012-2013 and who survived for a year following SNF admission. About 21% of these individuals were enrolled in an MA plan with a star rating of 4.5 or 5 (high star rating plans). Table 1 presents summary statistics of all included beneficiaries. The mean age of the patients in our sample is 80 years old; 66% of these patients are female. There are some key differences between enrollees in low and high star rating plans. Patients in highly rated plans are more likely to be white, non–dual-eligible and married than patients in low star rating MA plans. Enrollees in high star rating plans have lower prevalence of key diagnoses such as diabetes and Alzheimer’s disease.

**Table 1.** Baseline Characteristics and Outcomes of Included MA Beneficiaries.

	All patients (N = 529,962)	Patients enrolled in 3 to 4-star plans (n = 417,396)	Patients enrolled in 4.5 and 5-star plans (n = 112,566)
Patient characteristics			
Age	79.91	79.77	80.43
Female	66.24%	66.28%	66.07%
Race: Black	7.92%	8.83%	4.56%
Other race	6.93%	7.19%	5.97%
Fully dual-eligible	8.77%	9.53%	5.95%
Partially dual-eligible	5.03%	5.50%	3.27%
Married	39.40%	38.85%	41.47%
Activities of daily living total score (0-28, high = worse)	16.07	16.12	15.91
Baseline cognitive performance scale	1.14	1.15	1.12
Diagnosis indicators			
Stroke	8.88%	9.12%	8.00%
Lung disease	15.91%	16.07%	15.31%
Alzheimer’s disease	3.73%	3.85%	3.25%
Non-Alzheimer’s dementia	13.06%	13.44%	11.66%
Hip fracture	8.31%	8.38%	8.03%
Multiple sclerosis	0.30%	0.28%	0.39%
Heart failure	12.64%	12.61%	12.71%
Diabetes	29.27%	29.65%	27.86%
Schizophrenia	0.41%	0.43%	0.34%
Bipolar disease	1.11%	1.11%	1.12%
Aphasia	1.18%	1.20%	1.10%
Plan-SNF-level variables			
% of SNF’s patients enrolled in a patient’s MA plan	14.89	12.17	24.98
% of SNF county’s Medicare beneficiaries enrolled in a patient’s MA plan	9.19	7.76	14.44
Outcomes			
Any switching	21.19%	23.57%	12.37%
Switched to another MA plan	13.90%	15.42%	8.24%
Switched to traditional Medicare	7.30%	8.15%	4.13%

*Note.* MA = Medicare Advantage; SNF = skilled nursing facility.

**Figure 1.**
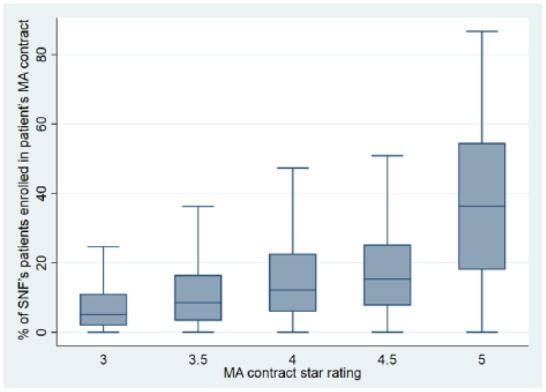
Box plots of SNF concentration experienced by enrollees, by MA plan star rating. *Note.* SNF = skilled nursing facility; MA = Medicare Advantage.

Table 1 also summarizes facility-level data. Specifically, it shows that for the average patient in a low star rating MA plan, the share of an SNF’s patients enrolled in their MA plan is fifteen percent. In contrast, the share of SNF’s patients enrolled in a high star rating plan is 25%. Figure 1 shows box plots of SNF concentration experienced by enrollees in different MA plans. For 3-star MA plans, the median level of SNF concentration was five percent. On the contrary, for 5-star MA plans, the median level of SNF concentration was 36%. Thus, enrollees in highly rated MA plans experienced much higher concentration.

**Figure 2.**
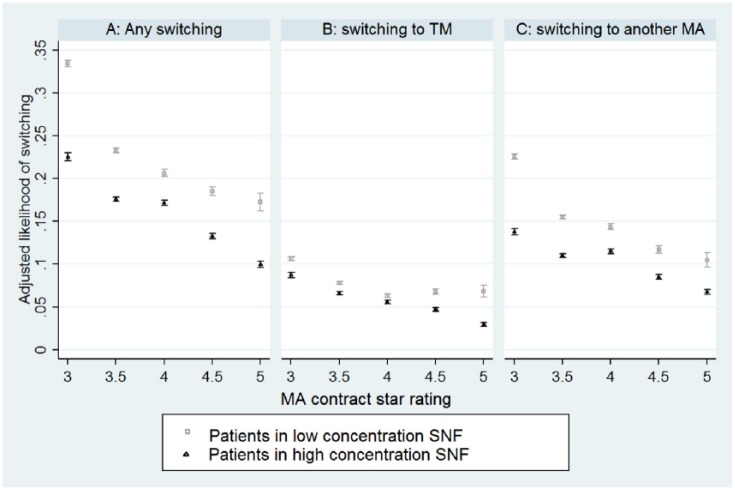
Likelihood of switching to another plan among MA enrollees treated in SNFs with low and high concentration of patient’s MA plan. *Note.* High-concentration SNF implies that the share of an SNF’s patients enrolled in a patient’s MA plan is greater than or equal to ten percent. These likelihoods are calculated adjusting for all patient characteristics listed in Table 1. MA = Medicare Advantage; SNF = skilled nursing facility; TM = traditional Medicare.

Figure 2 compares the likelihood of switching to another plan one year after SNF admission between patients admitted to high- and low-concentration SNFs. As noted, a high-concentration SNF implies at least ten percent of SNF’s patients were enrolled in the patient’s MA plan. We plotted three types of switching in three different panels: any switching either to another MA plan or to TM, switching only to another MA plan, and switching to TM. Patients in low-concentration SNFs have higher switching rates than patients in high-concentration SNFs. This remains true when comparing SNFs with similar star ratings. Of note, most patients in 5-star MA plans were admitted to high-concentration SNFs (see Figure 1). Yet the switching rates were consistently higher among patients in high-concentration SNFs with fairly tight confidence intervals.

Table 2 shows the regression estimates for switching outcomes among MA enrollees. These models were estimated as linear probability models including 2-way fixed effects: MA plan fixed effects and SNF fixed effects. For the entire sample, an increase in plan-specific MA concentration by ten percentage points is associated with a 0.3 percentage point decline in the likelihood of MA members switching to another plan (column 1). As shown in columns 2 and 3, 0.1 percentage points of this effect is due to decreased switching to TM and the remaining 0.2 percentage points is due to decreased switching to another MA plan. The categorized version of the concentration variable shows that compared with patients in SNFs with plan-specific MA concentration lower than ten percent, patients in SNFs with a concentration of ten percent to twenty percent have 3.5 percentage point lower likelihood and patients in SNFs with a concentration of twenty percent or more have a 5.6 percentage point lower likelihood of switching out from their original plan.

**Table 2.** Regression of Switching to Another Plan.

		(1)	(2)	(3)
		Any switching	Switching to another MA contract	Switching to TM
Alternative specifications				
Baseline specification (concentration continuous variable)		−0.00274***	−0.00178***	−0.000954***
		[–16.47]	[–13.37]	[–10.57]
Concentration as categorical variable (0-10 as reference category)				
10% ⩾ Concentration<20%		−0.035***	−0.026***	−0.0095***
		[–13.30]	[–11.04]	[–6.32]
Concentration ⩾20%		−0.056***	−0.038***	−0.0182***
		[13.27]	[–10.38]	[–7.70]
Alternative subsamples				
Stratifying variable	Sample			
Contract star rating	3-4-star contracts n = 413,227	−0.00351***	−0.00231***	−0.00120***
		[–15.39]	[–12.36]	[–9.956]
	4.5-5-star contracts n = 112,115	−0.00185***	−0.00137***	−0.000478**
		[–3.948]	[–3.417]	[–1.968]
SNF length of stay	Short-stay patients n = 443,845	−0.00223***	−0.00182***	−0.000418***
		[–13.21]	[–12.10]	[–5.934]
	Long-stay patients n = 81,487	−0.00348***	−0.00155***	−0.00193***
		[–9.055]	[–5.561]	[–5.770]
Dual-eligibility	Not dual-eligible n = 453,055	−0.00236***	−0.00166***	−0.000693***
		[–14.37]	[–11.64]	[–8.769]
	Dual-eligibles n = 72,277	−0.00424***	−0.00230***	−0.00194***
		[–10.66]	[–7.575]	[–6.017]

*Note.* Each coefficient and *t* stat is from a separate regression. All regressions include patient characteristics listed in Table 1, MA contract fixed effects, and SNF fixed effects. Square brackets report robust *t* statistics based on error clustered by SNFs. MA = Medicare Advantage; SNF = skilled nursing facility.

* *P* < .1 **P < .05 ***P < .01

Table 2 also presents the results of our subsample analyses, which indicate that the effect of concentration is higher for low star MA plans. The effect size is also larger among those who became long-stay residents following a SNF admission compared with short-stay residents (ie, those who leave a SNF after post-acute care). The effect size is higher for dual-eligible beneficiaries compared with nonduals. In general, this statistical association is very robust across all subsamples.

## Discussion

Using three years of Medicare data and examining MA beneficiaries newly admitted to SNFs, we find that highly rated MA plans steer their members to a smaller number of SNFs and this concentration is strongly associated with lower rates of switching out of the plan. We used a conservative model that takes both MA plan effects and SNF effects into account. Although our analysis cannot prove a causal relationship, these findings are consistent with the principle of economies of scale. That is, if a MA beneficiary is admitted to a SNF that frequently serves patients enrolled in his or her MA plan, the beneficiary is less likely to switch out of the plan in the next year than other patients in that plan and in that SNF.

Few studies have examined factors influencing the retention of high-cost, high-need populations, such as SNF users, in MA. Although modifications to how MA payments are risk-adjusted increased MA entry among beneficiaries in poorer health, these high-cost beneficiaries are still more likely to leave MA.^36^ Preliminary survey data from California’s efforts to enroll dual-eligible beneficiaries, including SNF users, in managed care plans suggest that beneficiaries may opt out of these plans because they are concerned about being able to access their physicians and other providers.^37,38^ Similar factors could influence disenrollment rates for the population of SNF users examined in our study.

One potential solution for MA plans to retain their patients is to form referral and care management relationships with particular health care providers or facilities that serve SNF populations. If a health care provider or facility has a large share of patients from a particular MA plan, then they might be more invested in building a care management relationship and be more adept at managing the billing requirements of that plan, leading to a better experience for plan members and improved plan retention. These findings are only valid if other SNF characteristics stay constant, including quality. In forming these referral networks, care must be taken to avoid excessively narrow networks (overly limiting patients’ choice of providers) and including high-quality SNFs in the network.

One possible explanation for why some SNFs have a greater share of patients from a particular MA plan is that SNFs may be more willing to accept patients from that MA plan. Several aspects of MA plan administration—payment rates, billing procedures, network restrictions, prior approval processes—may increase or decrease their attractiveness to SNFs. If SNFs are eager to accept patients from these plans, patients may also face fewer barriers from their plan for getting their stay covered. This may make them more likely to stay with their plan. In contrast, SNFs may be inappropriately requesting that their patients are disenrolled from MA plans. In response to complaints from SNF patients who were disenrolled from MA plans without their knowledge, CMS has warned SNFs that such practices are against CMS regulations and that a patients’ personal choice to disenroll from plans must be documented.^39^

Another finding is that a large share of SNF patients switch from one MA plan to another MA plan. There are frequent entries to and exits from MA plans that can trigger switching from one MA plan to another. In addition, because there is large variation in cost-sharing, plan benefits, and size/quality of provider networks, a patient may find another MA plan to be more appropriate based on the SNF care experience.^40-43^ Because we focused on MA enrollees who thought that MA would be more appropriate than TM to begin with, most of the MA beneficiaries remained in MA.

The main limitation of this work is that the relationship that we estimated shows statistical association and cannot be interpreted as a causal relationship. We control for SNF quality and practices via SNF fixed effects and MA plan quality via plan fixed effects, but other factors related to the local area environment may be influencing our outcome. More importantly, it is possible that MA plans are selectively steering their preferred patients to their frequently used SNFs and such steering can explain part of the association. However, we note that our patient population is fairly homogeneous. The association is also robust for patients with and without dual-eligibility and patients who had different lengths of stay (short vs long). Most importantly, SNF residents with long stays are more likely to switch to TM and exhibit a larger dose-response. This provides additional confidence that switching is not due to selective steering. Second, we have to infer network membership because we do not have the actual data on whether a particular SNF belonged to a specific network. Third, because our patients can have three discrete outcomes (not switching, switching to another MA plan, and switching to TM), a multinomial logit or probit model would have been appropriate. However, because we needed to include high-dimensional fixed effects (for ~14 000 SNFs and ~400 MA contracts), multinomial models become computationally very intensive and we had to use a linear probability model. We follow the approach of prior work that used linear probability models to examine switching from MA to TM.^8^ Finally, we do not know the payment rate or other limits on SNF length of stay that MA plans may impose on patients and SNFs. These may influence our ability to detect whether it was the SNF experience that “caused” patients to switch.

## Conclusions

Highly rated MA plans steer their patients to a smaller number of SNFs and these patients are less likely to switch out of their plans. Embracing selected SNFs as part of the network of preferred providers may decrease disenrollment rates for SNF beneficiaries and facilitate MA plans’ ability to continue to manage care for high-cost beneficiaries.
